# Sexual behavior and self-declaration of sexual orientation among people 18–64 years in Brazil: results from the Knowledge, Attitudes, and Practices survey, 2013 and the National Health Survey, 2019

**DOI:** 10.1186/s12889-023-16420-1

**Published:** 2023-08-02

**Authors:** Célia Landmann Szwarcwald, Ana Roberta Pati Pascom, Paulo Roberto Borges de Souza Júnior, Giseli Nogueira Damacena, Euclides Ayres Castilho

**Affiliations:** 1 Fundação Oswaldo Cruz, Instituto de Comunicação E Informação Científica E Tecnológica Em Saúde, Rio de Janeiro, RJ Brasil; 2 Ministério da Saúde, Secretaria de Vigilância Em Saúde, Brasília, DF Brasil; 3 Departamento de Medicina Preventiva, Faculdade de Medicina da Universidade de São Paulo, São Paulo, SP Brasil

**Keywords:** Self-declaration, Sexual orientation, Disclosure, Risk practices

## Abstract

**Background:**

Population surveys involving the monitoring of high-risk sexual behavior have been recognized as important public health tools to control the HIV epidemic and other sexually transmitted infections (STIs).

**Methods:**

Using data from the Knowledge, Attitudes, and Practices survey (PCAP-2013) and from the National Health Survey (PNS-2019), indicators of sexual behavior were compared according to sociodemographic characteristics among individuals aged 18–64 years, including size (%) estimates of men who have sex with men (MSM) and women who have sex with women (WSW). Specifically, the PNS-2019 prevalence estimates of homosexual, bisexual, heterosexual males and females were compared with those from the PCAP-2013. To compare PCAP and PNS proportional distributions, the Pearson's chi-square test, adjusted by the Rao-Scott’s correction, was applied.

**Results:**

Size (%) estimates of MSM and WSW obtained by direct questions from the PCAP-2013, showed higher homosexuality prevalence estimates than those resulting from the PNS-2019 self-declared sexual orientation. Significant differences were found between the MSM proportions according to the PCAP-2013 (3.7%; 95% CI 3.1–4.4%) and to the PNS-2019 (2.2%; 95% CI 1.9–2.5), and between the WSW proportions (4.6%; 95% CI 4.0–5.4%) and (2.1%; 95% CI 1.8–2.4), respectively. Results from both surveys showed MSM and WSW prevalence estimates increase with educational level, decrease with age, and is larger among people who do not live with partner, live in urban areas and in state capitals. Regarding condom use at last sexual intercourse, no differences between the PCAP-2013 and the PNS-2019 estimates were found at the national level, but significant improvements were found for MSM, people aged 18–24 and 25–34 years, and individuals not living with a partner.

**Conclusions:**

The underestimation of MSM and WSW prevalence by self-declared sexual orientation suggests that sexual minorities face many difficulties related to disclosing their sexuality and reinforces the importance of developing public health interventions for changing population attitudes and promoting sexual orientation disclosure. Moreover, the low use of condoms in both surveys (PCAP-2013 and PNS-2019) carried out 6 years apart highlights the need of public policies to expand prevention strategies for HIV infection and other STIs.

## Introduction

Population surveys involving the monitoring of risky sexual behavior have been recognized as important public health tools to control the HIV epidemic and other sexually transmitted infections (STIs). These studies help inform preventive measures by increasing the effectiveness of public health interventions [1, 2].

In Brazil, several initiatives were undertaken in the 2000s to monitor risky behaviors related to HIV infection. In 2004, the Division of AIDS, STI, and Viral Hepatitis of the Ministry of Health (MoH) with the support of the Centers of Disease Control Global Aids Program in Brazil (CDC GAP-Brazil) conducted a national survey on Knowledge, Attitude, and Practices related to HIV infection and other sexually transmitted infections, called PCAP—“Pesquisa de Comportamentos, Atitudes e Práticas”, in Portuguese [3].

The PCAP is a Behavioral Surveillance Survey (BSS) that enables the tracking of temporal and spatial trends in HIV knowledge, attitudes, and risk behaviors in selected groups within the Brazilian population. Periodic rounds of this survey (2008, 2013) provided an opportunity to supply information to develop specific indicators for the monitoring and evaluation of measures and prevention strategies [4–6].

Among the main survey objectives were those intended to describe STI risk practices according to sociodemographic characteristics, estimating the size of key populations and coverage of periodic HIV testing both in the general population and among high-risk groups for HIV infection [5]. Specifically, the PCAP aimed to monitor risk practices for sexually transmitted infections, analyze how health policies influence attitudes and practices, and collect information to support HIV prevention and control actions [7–9].

Another important behavioral survey in Brazil, with broader objectives than the PCAP, is the National Health Survey (PNS—“Pesquisa Nacional de Saúde” – in Portuguese). The PNS was carried out for the first time in 2013, based on three fundamental axes: the national health system’s performance, health conditions, and self-reported morbidity and associated risk factors [10].

The second edition of the PNS was carried out in 2019 and gave continuity to most of the modules covered in the first edition, involving an even larger sample of households [11]. New modules required by technical areas of the Ministry of Health (MoH) were included in the PNS-2019. Among these was a module focused on sexual activity and behavior [12]. One of the main questions was the self-declaration of sexual orientation.

In the present study, information on sexual behavior and the self-declaration of sexual orientation obtained in the PNS-2019 was compared to that obtained in the PCAP- 2013. Using data from both surveys, the indicators of sexual behavior were compared according to sociodemographic characteristics. The analysis of the results was carried out in light of the differences in the elaboration of the questions and in the sampling designs of the two surveys.

## Methods

### PCAP-2013

The PCAP is a cross-sectional study, conducted in a nationwide population-based household survey. The population surveyed consisted of residents in permanent private households (PPH) in Brazil, not including those located in special census tracts (quarters, military bases, accommodations, camps, vessels, penitentiaries, penal colonies, prisons, jails, asylums, orphanages, convents, and hospitals) [5]. The project was approved by the MoH Ethics Committee in February 2013 (logged under protocol number 194,434).

#### Sampling Design

The sample size of the PCAP-2013 was set at 12,000 individuals, aged 15- 64 years. The sample size was calculated to estimate the proportion of HIV testing over the 12 months prior to the survey (12%) with a two-sided error of 1.3% and a 95% confidence interval (CI).

The PCAP-2013 sample was selected using a 3-stage cluster sampling with stratification of the primary sampling units. The census tracts were considered the primary sampling units (PSU). The sampling design consisted of stratifying the primary sampling units by: Major Region (North, Northeast, Southeast, South, Center-West); population size of the municipality of residence (< 50,000; 50,001–200000; > 200,000 inhabitants); and type of household situation (rural/urban). Using these variables, 30 strata were created.

In each of the 30 strata, the PCAP sample was selected in 3 stages. In the first stage, a systematic sample of census tracts was selected from the Geographic Operational Base of the 2010 Demographic Census, using implicit stratification according to the educational level of the head of the household. Approximately 60 tracts were selected in the North Region, 200 in the Northeast Region, 320 in the Southeast Region, 110 in the South Region, and 60 in the Center-West Region. In the second stage, 16 households were randomly selected in each census tract.

In the third stage, in each household, only one resident, aged 15–64 years, was chosen for the interview. To represent all population subgroups of specific interest for the HIV epidemic (especially young people and men who have sex with men (MSM), the selection of the household resident sought to complete the quotas of the 3 variables: gender (Male/Female), age range (15–24; 25–34; 35–49; 50–64 years) and living with a partner or not.

#### Data weighing

Due to the oversampling of some population groups, the sample was calibrated according to the 2010 census distribution by Major Region, age group, living with a partner or not, and educational level. A limitation of this procedure, however, is the choice of variables to be included in the post-stratification procedure. The absence of variables associated with the outcome may affect the estimates of the indicators of interest [13].

### PNS-2013 and PNS-2019

The PNS is a nationwide population-based household survey conducted by the Brazilian Institute of Geography and Statistics (IBGE) in partnership with the MoH.

The PNS was carried out in 2013 and in PNS 2019. In the two PNS editions, the surveyed population includes Brazilian residents of private households, except those located in special census tracts.

The PNS-2013 was approved by the National Commission of Ethics in Research (CONEP) in June 2013 (Protocol No. 328,159) and the PNS-2019 in August 2019 (Protocol No. 3,529,376).

#### Sampling design

As part of the Integrated Household Survey System of the IBGE, the PNS sample is a subsample of the IBGE Master Sample. The PNS sample is selected with a 3-stage cluster sampling (census tracts, households, household residents) from the IBGE Master sample. In the three stages, the subsample is selected by simple random sampling, so the selection of primary sampling units (PSU) follows the same PSU stratification of the IBGE Master Sample. Further details of the PNS sampling process are available in a previous publication [11].

In 2013, the total sample was composed by 60,202 people aged 18 years or over, and in 2019, by 90,846 people aged 15 years or older.

#### Data weighing

The expansion factors were calculated by the inverse product of the selection probabilities in each stage. The expansion factors were calibrated, considering the population projection by Federation Units, sex, and age for 2010–2060 and an adjustment factor for losses.

In the present study, to compare the results of the PCAP-2013, PNS-2013 and PNS-2019, only individuals aged 18–64 years were considered in the three surveys.

### Study variables

a Population size (%) of homosexual, bisexual, heterosexual men and women: these indicators were estimated from the PCAP question “Do you currently have sexual intercourse: i) only with men? ii) with men and women? iii) only with women? Affirmative answers to items i) and ii) defined the proportion (%) of men who have sex with men (MSM) and item 3 established the proportion of heterosexual men. The same questions were used for women to establish the population size (%) of women who have sex with women (WSW). From the PNS, these indicators were based on the self-declaration of sexual orientation: (homosexual, bisexual, heterosexual, other, do not know, refused to answer) for both males and females. Due to the small number of respondents, the category “other” was omitted from the analysis. The categories “do not know” and “refused to answer” were considered missing values.
b Condom use at last sexual intercourse: From the PCAP, this indicator was estimated as the use of a condom at last sexual intercourse in the past 12 months with fixed or casual partners. From the PNS-2019, prevalence of the use of a condom at last sexual intercourse was based on a unique question: In the past twelve months, did you use a male or female condom at last sexual intercourse?
c Sociodemographic variables: Major Regions (North, Northeast, Southeast, South, Center-West); gender (Male/Female); age group (18–24, 25–34, 35–49, 50–64 years); educational level (up to complete elementary school, up to complete middle school, incomplete secondary school or higher); race/color (white/nonwhite); living with a partner (yes/no); and household situation (rural/urban).

### Statistical analysis

As the PCAP and the PNS use stratification of census tracts and multistage cluster selection, the complex sample designs of the three surveys were considered in all statistical analyses. Data were analyzed using the Software for Statistics and Data Science (Stata) version 14.0, “survey” module.

First, the PCAP-2013, PNS-2013, and PNS-2019 proportional distributions of individuals aged 18–64 years by sociodemographic variables (gender, age group, educational level, race/skin color, living with a partner or not, Major Region of residence, and household situation rural or urban) were compared. To test differences between the PCAP-2013 and PNS-2013 proportional distributions by sociodemographic variables, at the 5% level of significance, as well as to test differences between the PNS-2013 and PNS-2019 proportional distributions we used the Pearson’s chi- square test, adjusted by the Rao-Scott correction to account for the survey design effects.

Using data from the PCAP-2013 and the PNS-2019, prevalence of sexual behavior indicators and the corresponding 95% Confidence Intervals (95% CI) among individuals aged 18–64 years were estimated according to the sociodemographic variables. Specifically, the PNS-2019 prevalence estimates of homosexual, bisexual, heterosexual males and females were compared with those from the PCAP- 2013. As the PCAP MSM size (%) estimate is similar to that of the Joint United Nations Program on HIV/AIDS, UNAIDS for Latin America [14], and is in line with the international standard in countries that do not prohibit homosexuality [15, 16], the size estimates (%) of the PCAP sexual minorities were taken as the references to PNS-2019 estimates based on the self-declaration of sexual orientation. The Pearson’s chi-square test, adjusted by the Rao-Scott correction, was used to test prevalence differences at the 5% level of significance.

## Results

The PCAP-2013, PNS-2013, and PNS-2019 proportional distributions of individuals by sociodemographic characteristics are shown in Table 1. In the comparison of the two surveys conducted in 2013, minor differences were found for most variables, except for the distributions by gender, age-group, skin color, and educational level. Comparison of the two PNS editions showed significant differences for age-group, skin color, and educational level.

**Table 1:** Proportional (%) distributions of individuals aged 18–64 years by sociodemographic characteristics using data from PCAP^1^-2013, PNS^2^-2013, and PNS^2^-2019

**Characteristics**		**PCAP**^1^**-2013**		**PNS**^2^**-2013**		**PNS**^2^**-2019**	
		**%**	**CI (95%)**	**%**	**CI (95%)**	**%**	**CI (95%)**
**Region**	North	7.7	6.9–8.6	7.8	7.6–8.0	8.2	7.9–8.5
	Northeast	26.7	24.2–29.4	26.6	26.0–27.2	26.5	25.9–27.2
	Southeast	43.4	41.0–46.0	43.3	42.5–44.1	42.9	42.0–43.9
	South	14.8	13.5–16,1	14.7	14.2–15.2	14.5	14.1–15.0
	Center-West	7.4	6.5–8.3	7.6	7.4–7.8	7.8	7.5–8.2
**Gender**^**a**^	Male	49.2	48.8–49.5	47.6	46.8–48.4	47.5	46.9–48.2
	Female	50.8	50.5–51.2	52.4	51.6–53.2	52.5	51.8–53.1
**Age Group**^**a,b**^	18–24	18.5	18.0–19.1	18.2	17.5–18.8	16.3	15.7–16.9
	25–34	27.7	27.4–28.1	24.7	24.1–25.3	21.3	20.7–21.8
	35–49	32.7	32.3–33.1	32.2	31.5–32.9	34.4	33.8–35.1
	50–64	21.0	20.8–21.3	25.0	24.3–25.7	28.0	27.5–28.6
**Race/skin color**^**a,b**^	White	39.1	37.2–41.0	46.5	45.7–47.4	41.8	41.1–42.6
	Nonwhite	60.9	59.0–62.8	53.5	52.6–54.3	58.2	57.4–58.9
**Live with partner**	Yes	61.7	61.2–62.2	62.3	61.5–63.1	62.9	62.1–63.6
	No	38.3	37.8–38.8	37.7	36.9–38.5	37.1	36.4–37.9
**Educational level**^**a,b**^	Up to complete elementary school	33.8	32.3–35.3	33.8	33.0–34.7	28.9	28.2–29.6
	Up to complete middle school	22.6	21.5–23.8	16.7	16.1–17.2	15.6	15.1–16.1
	Secondary school or higher	43.6	42.0–45.2	49.5	48.6–50.4	55.5	54.7–56.3
**Household situation**	Urban	85.1	83.1–86.8	86.3	85.9–86.8	86.4	85.9–86.8
	Rural	14.9	13.2–16.9	13.6	13.2–14.1	13.6	13.2–14.1

^1^Knowledge, Attitudes, and Practices Survey; ^2^ National Health Survey

^a^Significant difference (*p* < 5%) between the PCAP-2013 and PNS-2013 proportional distributions

^b^Significant difference (*p* < 5%) between the PNS-2013 and PNS-2019 proportional distributions

Regarding the age distributions, the larger differences were found for the oldest age group (50–64 years), with proportions reaching 21% (PCAP-2013), 25% (PNS-2013), and 28% (PNS-2019). Significant differences in the educational level distributions were also found. The PCAP-2013 showed a proportion of 43.6% of people with a secondary education or higher. The PNS-2013 and PNS-2019 showed proportions of 49.5% and 55.0%, indicating improvements in the Brazilians’ educational level. As to race/skin color, the PCAP-2013 proportion found for white skin color seems to be underestimated, most likely because this variable was not considered in the PCAP calibration process. Variations in PNS black skin color proportions from 2013 to 2019 may be attributed to changes in black skin color self-identification, with a higher percentage of people declaring a black skin color in 2019 than in 2013 [17] (Table 1).

Table 2 presents the population sizes (%) of homosexual/bisexual males and females, obtained by direct questions from the PCAP-2013 and from the PNS-2019 self-declared sexual orientation. Significant differences were found between the MSM proportions according to the PCAP (3.7%; 95% CI 3.1–4.4%) and to the PNS (2.2%; 95% CI 1.9–2.5), as well as between the WSW proportions: (4.6%; 95% CI 4.0–5.4%) and (2.1%; 95% CI 1.8–2.4), respectively.

**Table 2:** Proportion (%) of homosexual and bisexual individuals aged 18–64 years by sex. PCAP-2013 and PNS-2019

**Category**		**PCAP-2013**^a^		**PNS-2019**^b^	
		**%**	**95% CI**	**%**	**95% CI**
**Male**	Sex with men and women	1.3	0.9–1.5	0.6	0.4–0.7
	Sex only with men	2.4	1.9–3.0	1.6	1.4–1.9
	MSM*	3.7	3.1–4.4	2.2	1.9–2.5
**Female**	Sex with men and women	3.4	3.1–3.8	1.0	0.8–1.2
	Sex only with women	1.2	0.9–1.7	1.1	0.9–1.3
	WSW*	4.6	4.0–5.4	2.1	1.8–2.4

^a^Knowledge, Attitudes, and Practices Survey: Based on the direct question: “Do you currently have sexual intercourse: i) only with men? ii) with men and women? iii) only with women?

^b^National Health Survey: Based on self-declaration of sexual orientation

MSM – men who have sex with men

WSW – women who have sex with women

^*^Significant difference (*p* < 5%) between the PCAP-2013 and PNS-2019 proportions

Table 3 shows the MSM size (%) estimates according to sociodemographic characteristics. For all sociodemographic characteristics, the PCAP-2013 MSM size (%) estimates were higher than PNS-2019 estimates, but not always significantly higher. The highest MSM proportions were found among men aged 18–24 years in both surveys, achieving 6.3% (95% CI: 4.5–8.9%) and 5.2% (95% CI: 4.0–6.7%), respectively, and the difference between the estimates was not statistically significant at the 5% level. Among men who live in a state capital, there was no significant difference in the two survey estimates: 3.6% (95% CI: 3.0–4.3%) by the PNS-2019 and 4.0% (95% CI: 2.7–6.1%) by the PCAP-2013. Regarding differences by Major Region, no significant differences were found in the Northeast, South, and Center-West. In the Southeast Region, the PCAP MSM size (%) was significantly higher than the PNS estimate, but this region showed the highest MSM prevalence in both surveys.

**Table 3:** Prevalence (%) of MSM aged 18–64 years by sociodemographic characteristics. PCAP^1^- 2013 and PNS^2^-2019

**Characteristics**	**Category**	**PCAP-2013**^a^		**PNS-2019**^b^	
		**%**	**95% CI**	**%**	**95% CI**
**Total**	Brazil^*^	3.7	3.0–4.5	2.2	2.0–2.6
**Region**	North^*^	5.6	3.9–8.0	2.4	1.7–3.6
	Northeast	2.3	1.4–3.8	2.0	1.6–2.5
	Southeast^*^	4.9	3.7–6.6	2.5	2.0–3.2
	South	2.0	1.2–3.5	1.9	1.5–2.5
	Center-West	2.9	1.8–4.8	1.9	1.5–2.5
**Age Group**	18–24	6.3	4.5–8.9	5.2	4.0–6.7
	25–34^*^	4.1	3.0–5.5	2.6	2.2–3.2
	35–49^*^	2.8	2.0–3.9	1.7	1.3–2.2
	50–64^*^	2.1	1.3–3.2	0.8	0.6–1.2
**Race/skin color**	White	2.7	2.0–3.8	2.0	1.6–2.5
	Nonwhite^*^	4.2	3.3–5.3	2.4	2.0–2.9
**Live with partner**	Yes^*^	2.2	1.6–2.9	0.8	0.6–1.0
	No	6.1	4.8–7.9	5.3	4.5–6.2
**Educational level**	Up to complete elementary school^*^	2.1	1.4–3.1	0.7	0.5–1.0
	Up to complete middle school	4.2	2.9–5.9	2.8	1.9–4.1
	Secondary school or higher^*^	4.6	3.5–6.0	3.0	2.6–3.4
**Household situation**	Urban^*^	3.9	3.2–4.9	2.5	2.1–2.8
	Rural	2.4	1.2–4.5	1.1	0.7–1.7
**Live in a state capital**	Yes	4.0	2.7–6.1	3.6	3.0–4.3
	No^*^	3.6	2.8–4.5	1.8	1.5–2.2

^a^Knowledge, Attitudes, and Practices Survey: Based on the direct question: “Do you currently have sexual intercourse: i) only with men? ii) with men and women? iii) only with women?

^b^National Health Survey: Based on self-declaration of sexual orientation

MSM – men who have sex with men

^*^Significant difference (*p* < 5%) between the PCAP-2013 and PNS-2019 proportions

Regarding the educational level, although significant differences were found in the comparison between the two survey results, as the level of education improves, the homosexuality size estimates increased. Based on the PCAP-2013 results, the MSM size estimate ranged from 2.6% to 4.1%, and based on PNS, from 0.7% to 3%, from up to complete elementary school to secondary school or higher. The analysis by skin color also showed significant differences between the two survey estimates, but MSM proportions were higher among non-white males, in both the PCAP-2013 and the PNS-2019. Moreover, significant differences were found for residents of urban areas.

The WSW size (%) estimates by sociodemographic characteristics according to PCAP-2013 and PNS-2019 are presented in Table 4. For almost all sociodemographic characteristics, the WSW size (%) estimates based on same-sex sexual intercourse were significantly higher than the estimates by self-declaration. Only for residents in the North and South regions and for women aged 18–24 years, there was no significant difference at the 5% level of significance. The highest WSW size (%) estimates were presented by women aged 18–24 years in both surveys, reaching 6.7% (95% CI: 4.7–9.5%) in PCAP-2013 and 6.1% (95% CI: 4.0–6.7%) in PNS-2019, followed by women who do not live with a partner, 6,6% (95% CI: 5.2–8.3%) and 3.4% (95% CI: 2.8–4.0%) respectively, and women who live in a state capital, 5.7% (95% CI: 4.5–7.2%) and 3.0% (95% CI: 2.5–3.6%) respectively. Besides, results from both surveys show WSW prevalence increases with educational level, decreases with age, and is larger among women who do not live with partner, live in urban areas and in state capitals.

**Table 4:** Prevalence (%) of WSW by sociodemographic characteristics. PCAP^1^- 2013 and PNS^2^-2019

**Characteristics**	**Category**	**PCAP-2013**^a^		**PNS-2019**^b^	
		**%**	**95% CI**	**%**	**95% CI**
**Total**	Brazil*	4.6	3.9–5.6	2.1	1.8–2.4
**Region**	North	3.2	1.9–5.5	1.8	1.4–2.4
	Northeast*	4.5	3.1–6.5	1.5	1.2–1.9
	Southeast*	5.3	4.2–6.8	2.4	1.9–3.0
	South	3.0	2.0–4.6	2.6	2.0–3.3
	Center-West*	5.7	3.9–8.2	1.9	1.4–2.6
**Age Group**	18–24	6.7	4.7–9.5	6.1	4.9–7.6
	25–34*	5.9	4.1–7.8	2.7	2.2–3.3
	35–49*	4.0	3.0–5.3	1.2	1.0–1.6
	50–64*	2.4	1.4–3.8	0.5	0.4–0.8
**Race/skin color**	White*	4.1	3.1–5.3	2.3	1.9–2.8
	Nonwhite*	5.1	4.1–6.2	2.0	1.6–2.4
**Live with partner**	Yes*	3.4	2.7–4.3	1.2	1.0–1.5
	No*	6.6	5.2–8.3	3.4	2.8–4.0
**Educational level**	Up to complete elementary school*	3.1	2.2–4.5	0.5	0.4–0.8
	Up to complete middle school	4.9	3.5–6.8	2.9	1.9–4.3
	Secondary school or higher*	5.7	4.5–7.2	2.5	2.1–3.0
**Household situation**	Urban*	4.9	4.0–5.9	2.3	2.0–2.6
	Rural*	3.1	1.9–5.1	0.9	0.5–1.5
**Live in a state capital**	Yes*	5.7	4.1–7.8	3.0	2.5–3.6
	No*	4.3	3.4–5.1	1.8	1.5–2.1

^1^Knowledge, Attitudes, and Practices Survey: Based on the direct question: “Do you currently have sexual intercourse: i) only with men? ii) with men and women? iii) only with women?

^2^National Health Survey: Based on self-declaration of sexual orientation

WSW – women who have sex with women

^*^Significant difference (*p* < 5%) between the PCAP-2013 and PNS-2019 proportions

The analysis of the sexual behavior indicator “condom use at last sexual intercourse” by sociodemographic characteristics is presented in Table 5. At the national and regional levels, there were no differences between the PCAP and PNS estimates, meaning there was no increase in condom use at last sexual intercourse from 2013 to 2019. However, improvements were observed in some specific groups: individuals aged 18–24 years (from 58.7 to 67.2%) and 25–34 years (from 41.7 to 46.2%); not living with a partner (from 62.5 to 72.1%); and among MSM (from 66.3 to 80.4%), with the highest proportions of condom use at last sexual intercourse.

**Table 5:** Condom use at last sexual intercourse by sociodemographic characteristics among individuals aged 18–64 years. PCAP^1^-2013 and PNS^2^-2019

Characteristics		Condom use at last intercourse			
		**PCAP-2013**^a^		**PNS-2019**^b^	
		**%**	**95%CI**	**%**	**95%CI**
**Brazil**		**37.7**	**36.3–39.0**	**38.7**	**37.9–39.5**
**Region**	North	43.6	39.4–47.8	47.0	45.2–48.7
	Northeast^*^	35.8	33.5–38.1	39.5	38.4–40.7
	Southeast	37.7	35.3–40.3	38.1	36.7–39.6
	South	36.7	34.0–39.5	35.7	34.1–37.4
	Center-West	39.7	36.5–43.0	36.4	34.6–38.4
**Gender**	Male	42.9	41.2–44.6	41.0	40.0–42.1
	MSM^*^	66.3	57.3–74.3	80.4	74.5–85.1
	Heterosexual^*^	42.0	40.3–43.8	39.7	38.7–40.8
	Female^*^	32.1	30.2–34.1	36.3	35.3–37.4
**Age Group**	18–24^*^	58.7	55.7–61.6	67.2	65.1–69.3
	25–34^*^	41.7	39.4–44.0	46.2	44.6–47.8
	35–49	31.8	29.7–34.0	33.0	31.9–34.1
	50–64	20.4	18.3–22.6	21.6	20.5–22.8
**Race/skin color**	White	37.2	35.1–39.4	36.9	35.8–38.0
	Nonwhite^*^	37.9	36.3–39.6	40.1	39.1–41.0
**Live with partner**	Yes	25.8	24.3–27.3	25.5	24.8–26.3
	No^*^	62.5	60.1–64.7	72.1	70.8–73.5
**Educational level**	Up to complete elementary school	27.7	25.7–29.8	29.7	28.6–30.9
	Up to complete middle school	40.3	37.5–43.2	41.0	39.2–42.8
	Secondary school or more	43.2	41.2–45.3	42.2	41.1–43.2
**Household situation**	Urban	38.3	36.8–39.8	39.7	38.8–40.5
	Rural	34.1	30.9–37.6	32.7	31.4–34.0

^a^Knowledge, Attitudes, and Practices Survey: estimated as the use of condom at last sexual intercourse in the past 12 months with fixed or casual partners

^b^National Health Survey: Based on the question: In the past twelve months, at the last sexual intercourse you had, did you use a male or female condom?

MSM – men who have sex with men

^*^Significant difference (*p* < 5%) between the PCAP-2013 and PNS-2019 proportions

## Discussion

In the present study, we considered the comparability of data from the three surveys (PCAP-2013, PNS-2013 and PNS-2019) for a better comprehension of the differences in the estimated sexual behavior indicators.

In Brazil, since the PNS is a broad population-based household survey, conducted with a probabilistic sample of the Brazilian population in all stages, it is considered the gold standard of national health surveys [12]. Although the PCAP selection of individuals in the final stage (households) obeys a quota system, the comparison of the PCAP-2013 and the PNS-2013 distributions of individuals by sociodemographic characteristics showed minor differences for most of the study variables. Significant differences were found for the distributions by gender, age-group, educational level, and skin color. Comparison of the two PNS editions showed significant differences for the same sociodemographic variables, except for gender. As the PCAP-2013 was weighted according to the 2010 Demographic Census, and weighting of both PNS databases was based on natural expansion factors, these findings represent the aging of the Brazilian population from 2010 to 2019 and the improvement in Brazilians’ educational level [18].

Variations in PNS black skin color proportions from 2013 to 2019 have been discussed before and have been attributed to changes in the self-identification of black skin color [19]. However, the underestimation of the proportion of white people in the PCAP-2013 is most likely due to a statistical estimation problem, as this variable was not included in the survey post-stratification procedure [20].

Estimating the size and understanding diversity of homosexuality are essential for the development of inclusive policies and services [21]. To date, research on this topic is still scarce in Brazil. In 2019, the PNS has included the question of sexual orientation by self-declaration for the first time. The comparison with the PCAP-2013 direct questions about who they currently have sex with (men, women, or women and men) showed lower prevalence estimates of homosexuality based on sexual orientation self-declaration, for males and females, even though this questionnaire module is self-completed by the participants in both surveys.

Evidence that the use of different dimensions of sexual orientation (self-declaration and sexual behavior) generates different estimates of homosexuality prevalence was also found in a British study. While the proportions of homosexual/bisexual identity were 2.5% and 2.4% between men and women, 5.5% of men and 6.1% of women reported having sex with partners of the same sex. Furthermore, 28% of MSM and 45% of WSW identified themselves as heterosexual [22].

In addition, as the PNS-2019 question on sexual orientation self-declaration included only a few categories, individuals who did not feel comprised in any of the listed categories may not have responded adequately. Qualitative research on the best way to question sexual orientation recommended the use of multiple categories (lesbian, gay, homosexual, bisexual, heterosexual, transgender, etc.) with the possibility of selecting multiple options [23].

It is well-known that sexual minorities experience stigma and discrimination worldwide [24–26]. In Brazil, the underestimation of MSM and WSW size estimates based on the self-declared sexual orientation suggests that gay and bisexual communities experience various psychological challenges related to their sexuality and gender identity disclosure [27]. A qualitative study in Fortaleza, Brazil, indicated that MSM avoided seeking health care services due to a fear of being stigmatized and discriminated against. Furthermore, when they sought out health care, they tended to demonstrate masculine behavior so as not to be identified by their sexual orientation [28].

Additionally, it is important to consider the context in which the sexual orientation by self-declaration was asked, especially in terms of social acceptability. In 2019, year in which the PNS was conducted, an ultra-conservative agenda was implemented by the federal government, progressively decreasing support for diversity and human rights. This political scenario directly affected sexual and gender minorities in order to influence or even intimidate the self-declaration of sexual orientation [29].

For some specific population groups, the MSM size estimates showed higher differences between the PNS-2019 and the PCAP-2013 estimates. Findings revealed that men with incomplete fundamental schooling, living with a partner, residents in the North, aged 35 years and over, and living in the rural area or in cities other than state capitals were more likely not to reveal their sexual orientation. These results reinforce the importance of monitoring sexual orientation by self-declaration to continuously improve the quality of this information at subnational levels.

Identifying factors associated with the nondisclosure of sexual orientation is crucial for the development of interventions to strengthen protection from stigma and discrimination, as well as improve healthcare access among MSM. A survey in Germany showed that respondents who had never tested for HIV were more likely to live in a city with less than 100,000 inhabitants and to be less open about their sexual orientation to their primary care provider [30].

The indicator “condom use at last recent sexual intercourse” has the advantage of being questioned in the same way in both surveys. At the national level, no significant difference was found in the PNS-2019 when compared to the PCAP-2013, indicating that there is no general improvement in this behavior. However, the comparative analysis by sociodemographic variables showed significant increases for younger age groups (18–24 and 25–34 years), for MSM, and for those who do not live with a partner. These results show a greater awareness concerning HIV risk exposure in the most- at-risk population groups, suggesting that prevention actions focused on key populations have been successful [31].

However, although condom use is key to HIV prevention if used consistently and correctly, there are various barriers to regular condom use. The PNS-2019 data showed the main reason for not having used a condom at last sexual intercourse was trusting the sexual partner. The second reason most cited was the aversion to using a condom, more frequently cited among people with a low educational level. A recent systematic review revealed homosexual men believe that not using condoms represents mutual trust and loyalty between sexual partners, while condom use is a symbol of distrust or suspicion regarding the partner’s HIV status [32].

## Limitations

Among the limitations of this study, differences in the PCAP-2013 and PNS-2019 sample composition may be partly responsible for the variation in size (%) estimates of sexual minorities at the national level. However, the MSM and WSW prevalence estimates by sociodemographic characteristics are little affected, since they are estimates of conditional probabilities.

Regarding the PCAP-2013 and PNS-2019 differences in the sample designs, the former has a smaller sample size and larger design effects due to the high number of interviews in some clusters (census tracts). Thus, the large variance in the PCAP prevalence estimates may have affected the results of the statistical tests for comparing proportions.

In addition, differences in the main objective of the surveys may also explain variations in estimates. As the PNS-2019 is a general national health survey, respondents may have felt more comfortable answering this type of question in the PCAP-2013, specifically aimed at monitoring sexual attitudes and practices.

Another important limitation refers to the different ways of questioning sexual orientation in the two surveys, which may be considered the primary factor affecting the homosexual/bisexual prevalence estimates [33].

An additional limitation of this study lies in the difficulty to estimate the size of sexual and gender minorities. Although the PCAP-2013 MSM size (%) estimate in the PCAP is similar to that of Joint United Nations Program on HIV/AIDS, UNAIDS for Latin America [14], and is in line with the international standards in countries that do not prohibit homosexuality [15, 16], it is possible that the size of the MSM population is still underestimated [34, 35].

## Conclusions

The results of this study showed a large variation in the size of sexual minorities depending on the dimensions applied (sexual behavior or self-declaration). The underestimation of MSM and WSW prevalence by self-declared sexual orientation suggests that sexual minorities face many difficulties related to disclosing their sexuality and reinforces the importance of developing public health interventions for changing population attitudes and promoting sexual orientation disclosure. Moreover, the low use of condoms in both surveys (PCAP-2013 and PNS-2019) carried out 6 years apart highlights the need of public policies to expand prevention strategies for HIV infection and other STIs.

## Data Availability

The PNS databases are publicly available at the internet (https://www.pns.icict.fiocruz.br/bases-de-dados/). The PCAP database is available only upon request to the Departamento de HIV/Aids, Tuberculose, Hepatites Virais e Infeccões Sexualmente Transmissíveis, MS (https://www.gov.br/aids/pt-br) or to the author Ana Roberta Pati Pascom (ana.roberta@aids.gov.br) on reasonable request.

## Abbreviations

AIDS: Acquired Immuno deficiency Syndrome
BSS: Behavioral Surveillance Survey
CDC GAP-Brazil: Centers of Disease Control Global Aids Program in Brazil
CI: Confidence interval
CONEP: National Commission of Ethics in Research
HIV: Human immunodeficiency virus
IBGE: Brazilian Institute of Geography and Statistics
MoH: Ministry of Health
MSM: Men who have sex with men
PCAP: Knowledge, Attitudes and Practices survey
PNS: National Health Survey
PPH: Permanent private households
PSU: Primary sampling units
Stata: Software for Statistics and Data Science
STIs: Sexually transmitted infections
UNAIDS: Joint United Nations Program on HIV/AIDS
WSW: Women who have sex with women
